# A stable gene set for prediction of prognosis and efficacy of chemotherapy in gastric cancer

**DOI:** 10.1186/s12885-021-08444-w

**Published:** 2021-06-10

**Authors:** Rui Wu, Sixuan Guo, Shuhui Lai, Guixing Pan, Linyi Zhang, Huanbing Liu

**Affiliations:** 1 The First Affiliated Hospital of Nanchang University, Nanchang, Jiangxi China; 2 The Second Clinical College, Medical College of Nanchang University, Nanchang, Jiangxi China; 3 The First Clinical College, Medical College of Nanchang University, Nanchang, Jiangxi China; 4 Shangrao Maternity and Child Care Hospital, Shangrao, Jiangxi China; 5 School of Ophthalmology & Optometry, Nanchang University, Nanchang, Jiangxi China

**Keywords:** Gastric cancer, Molecular typing, Prognosis, Prediction of efficacy of chemotherapy, Immune infiltration

## Abstract

**Background:**

Gastric cancer (GC) is a primary reason for cancer death in the world. At present, GC has become a public health issue urgently to be solved to. Prediction of prognosis is critical to the development of clinical treatment regimens. This work aimed to construct the stable gene set for guiding GC diagnosis and treatment in clinic.

**Methods:**

A public microarray dataset of TCGA providing clinical information was obtained. Dimensionality reduction was carried out by selection operator regression on the stable prognostic genes discovered through the bootstrap approach as well as survival analysis.

**Findings:**

A total of 2 prognostic models were built, respectively designated as stable gene risk scores of OS (SGRS-OS) and stable gene risk scores of PFI (SGRS-PFI) consisting of 18 and 21 genes. The SGRS set potently predicted the overall survival (OS) along with progression-free interval (PFI) by means of univariate as well as multivariate analysis, using the specific risk scores formula. Relative to the TNM classification system, the SGRS set exhibited apparently higher predicting ability. Moreover, it was suggested that, patients who had increased SGRS were associated with poor chemotherapeutic outcomes.

**Interpretation:**

The SGRS set constructed in this study potentially serves as the efficient approach for predicting GC patient survival and guiding their treatment.

**Supplementary Information:**

The online version contains supplementary material available at 10.1186/s12885-021-08444-w.

## Introduction

Gastric cancer (GC) ranks the 6th place in terms of cancer morbidity, and it is also the 5th cause of cancer deaths in the world [1]. The overall survival (OS) rate of GC cannot be improved through surgery or neoadjuvant therapy [2]. GC is a kind of heterogeneous malignant tumor, whose primary or acquired drug resistance makes chemotherapy unable to completely destroy tumor cells, while insensitivity to chemotherapy is a common cause of tumor recurrence and metastasis [3]. Therefore, the evaluation of the overall survival, progression-free interval (PFI) and chemotherapy effect of patients with GC can help optimize the treatment strategy. The development of clinical prediction model is a conventional method to predict prognosis, and the key of modeling lines in the selection of stable and effective variables.

Conventional clinicopathologic variables, such as depth of invasion (T Stage) or lymph node metastasis (N stage), are predominantly focused on cancer cells to predict prognosis. While these variables are valid and widely used, they do not provide sufficient prediction [4]. Before this study, some articles have proposed new factors in addition to clinical factors for predicting the prognosis of GC, but the area under the curve (AUC) of the prediction model is not high, which suggests that new, more effective predictors need to be discovered [5].

DNA microarray technology or “gene chips,” derived from large-scale sequencing methods, are increasingly used to produce much more data than represents the sequence itself. It sheds novel lights on the pathophysiology and classification of disease, gene function, as well as drug research [6]. Using DNA microarray technology, we developed a reliable prognostic gene set in the hope of predicting overall survival, progression-free interval (PFI) and the chemotherapeutic effects on GC cases, thus laying solid foundation for treatment in clinic.

## Materials and methods

### Transcriptome data acquisition and clinical information collection

The Cancer Genome Atlas (TCGA) provides a large, free reference database for cancer research through the collection of cancer-related omics data, which is publicly available at the Data Portal TCGA (https://cancergenome.nih.gov). We downloaded the expression matrix of GC patients and relevant clinical information from the TCGA database in September 2018. The clinical information included overall survival, progression-free interval, AJCC pathologic tumor stage, histologic grade, gender and age.

### Study population and clinicopathological variables

We used the “createDataPartition” package in R to divided the data set into training cohort and validation cohort according to the stage stratified sampling with a ratio of 7:3. In this study, we used two analysis endpoints: OS, the time interval from diagnosis to death; PFI, the time interval between the beginning of observation and tumor progression.

### Stable prognostic gene identification and selection

In order to obtain stable prognostic genes, bootstrapping testing was used to test the stability of the initial genes. Seventy percent of patients were randomly selected from samples to assess the genetic impact on survival. After 1000 iterations, genes enrolled into 70% resampled runs (*P* < 0·05 upon stability test) were selected to be the creditable prognostic genes. Survival analysis was performed on all patients using the R software, and the genes with *P* value less than 10e-3 were screened for further study. The genes selected out after these two steps were identified as stable prognostic genes.

### Gene set generation using LASSO cox regression

LASSO regression is a statistical method that can not only select variables but also make regularization [7]. In biological and medical research, it is also used to build prediction models in data sets with many interrelated independent variables [8]. Therefore, LASSO regression has important statistical characteristics that help to assess the relationship between many biomarkers and clinical characteristics [9]. Using LASSO regression, select ten-fold cross validation, intercept the modeled optimal penalty parameter value, and finally generate the optimal genetic set for predicting prognosis. Based on the generated gene set, we used Cox analysis to obtain the risk scores of OS and PFI with OS and PFI as endpoint variables, respectively. Risk scores for each patient will be generated using the following formula:

$$ \mathrm{Risk}\ \mathrm{scores}=\sum \limits_{i=1}^n\beta i\times i $$

*βi* referred to the coefficients of each gene; *i* represented the expression value of the gene; n was the number of genes selected.

### Estimation of immune infiltration

Tumor is a kind of tissue with high heterogeneity, where the tumor microenvironment (TME) surrounds and interacts with the malignant cells, and the TME contains various immunocyte types. The dialectical relationship of cancer cells with immune microenvironment is of critical clinical significance; therefore, it is necessary to develop approaches to investigate the cell components in immune microenvironment [10]. MCP-counter package from the R software might be used in this case, which using the gene expression matrix to produce the scores of immunocytes (T cells, CD8 T cells, cytotoxic lymphocytes, B cells, NK cells, monocytes, dendritic cells, neutrophils, endothelial cells and fibroblasts). The MCP-counter estimates represented scores of individual samples because they are calculated independently from each sample [11]. The MCP-counter package of R software was adopted for converting the mRNA data to non-tumor cell infiltrating levels within TME. Before the analysis by MCP-counter, the standard annotation file was used to make the gene expression profile.

### Gene set variation analysis (GSVA)

GSVA calculates the enrichment fraction of the sample gene set according to the gene function inside and outside the gene set, which is a non-parametric, non-supervised competitive gene set test. Conceptually, such method may be interpreted to alter the gene expression data coordinate system from one gene to one gene set [12]. To assess pathway variability in large heterogeneous populations with complex phenotypic characteristics, we applied RNA-seq data and GMT to GSVA and acquired the enrichment fraction of each sample.

### Immunohistochemistry

Immunohistochemistry was obtained from the human protein atlas (HPA) (http://www.proteinatlas.org/) [13]. The expression levels of different expression genes, which chosen to build the OS and PFI models, were evaluated between normal stomach tissues and GC tissues from the HPA.

### Statistical analysis

The survival rate was calculated by the Kaplan-Meier method, while significance of difference was determined by log-rank test. Cox proportional hazard models with the stepwise method “LRforward” were used for single factor and multiple factor analysis. The Iasonos’ guide was used to construct and validate the nomogram [14]. The accuracy of survival prediction of the prognostic model was evaluated by time-dependent ROC as well as the Harrell’s concordance index (c-index). R package was employed for statistical analysis and *P* value were tested by double-tail. The truncation points of *P* values were statistically significant.

## Results

### GC patients’ characteristics and stable prognostic gene identification

The detailed characteristics of the patients in this study are as follows (Supplemental Table S1). In this study, 362 patients with clinical information in the TCGA data set were screened. The mean age at diagnosis was 67.0 years (range:30.0–90 years), 234 (64.6%) were males, and 128 (35.4%) were females. All patients screened had OS and PFI information. The mean survival days of OS was 603.7 days, and the mean survival days of PFI was 543.6 days. Through bootstrapping testing described in the materials, 1446 genes were screened. After survival analysis, 425 of the 1446 genes were screened. Because these 425 genes had at least 70% chance of being associated with survival in 1000 iterations, and *P* < 1e-3 was found in the survival analysis of all samples, they were identified as stable prognostic genes (Supplemental Table S2).

### Construction of molecular subgroups using stable prognostic genes

**Fig. 1:**
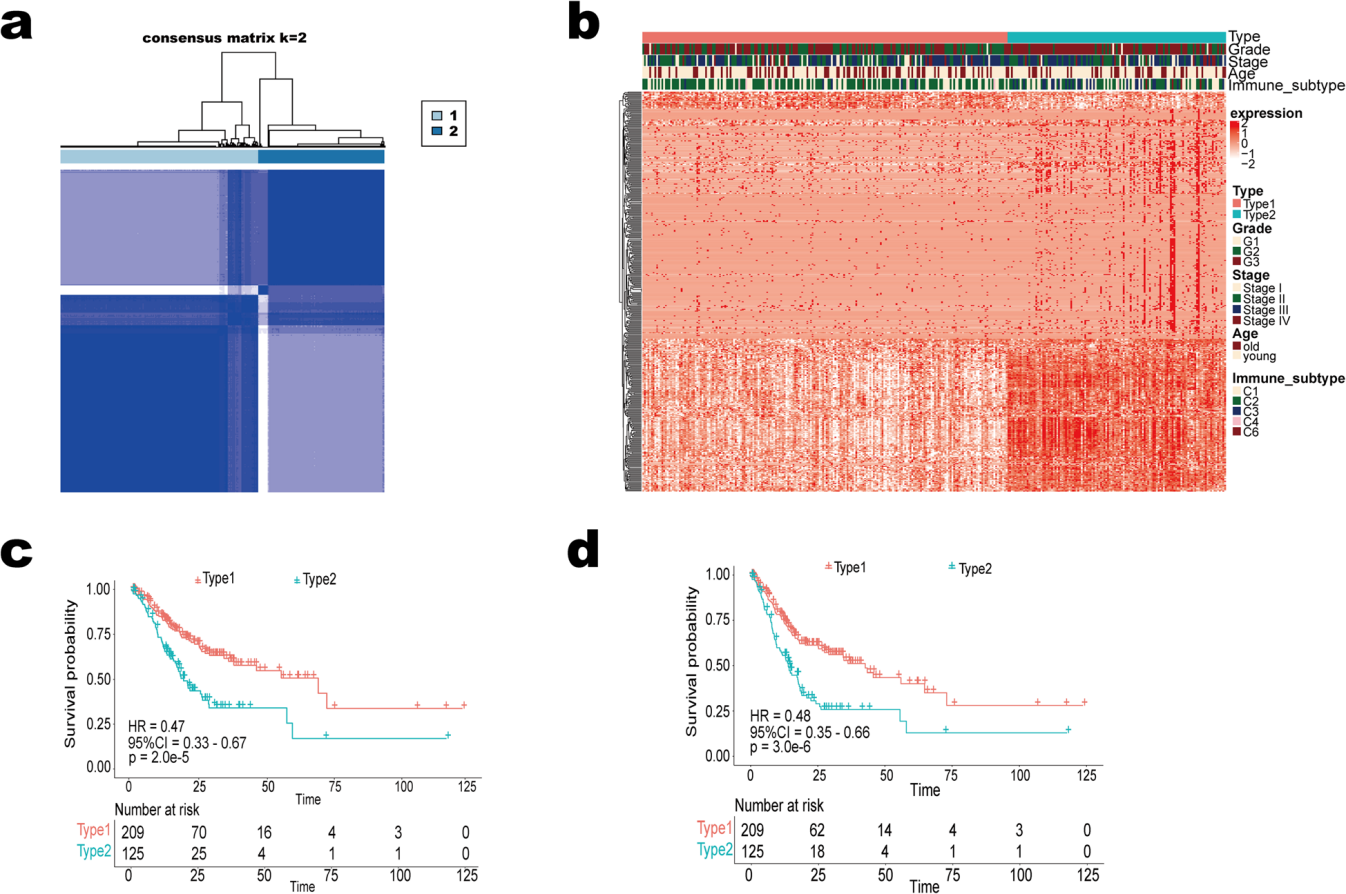
Consensus clustering of stable prognostic genes in gastric cancer. **a** Consensus matrices of gastric cancer patients(k = 2); **b** Gastric cancer cases are divided into two subtypes based on unsupervised analysis and hierarchical clustering of 425 stable prognostic genes. Clinical information (AJCC pathologic tumor stage, histologic grade, gender), immune subtype, and type are indicated above the heatmap; **c**–**d** Differences of patient overall survival **c** and progression-free interval **d** with two clusters

Unsupervised clustering was adopted for classifying GC to diverse molecular subtypes on the basis of those 425 stable prognostic genes with the R package “ConsensusClusterPlus”. We divided the patients into two types through the package (Fig. 1a-b). Kaplan-Meier curve showed there was a significant difference in OS and PFI between the two types of patients (Fig. 1c-d). The patients with better OS and PFI were redefined as Type1, and patients with poorer OS and PFI were defined as Type2. The patients of Type2 significantly had a more advanced grade compared with Type1 (Supplemental Table S3).

### Exploration of TME in type 1 and type 2 patients

**Fig. 2:**
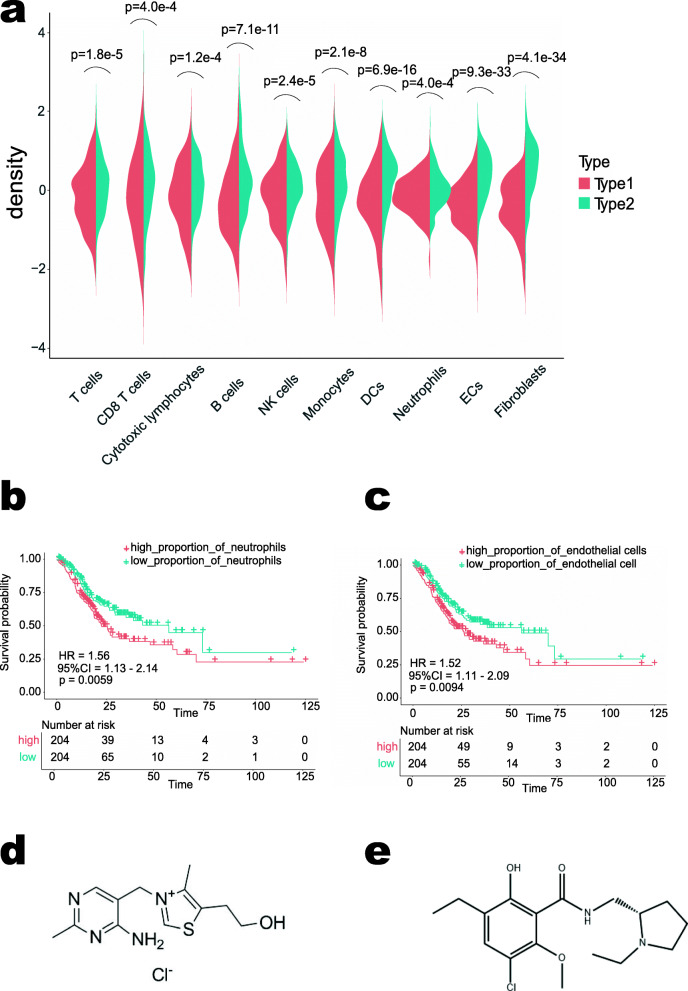
Exploration of tumor microenvironment in Type 1 and Type 2 patients. **a** Violin plot of the comparison of immune and stromal cell infiltration between the two types; (**b**-**c**) Kaplan–Meier curves of overall survival according to the cell infiltrating scores of neutrophils (**b**) and endothelium (**c**). **d**-**e** Connectivity Map (CMap) analysis results; chemical structure of thiamine (**d**); chemical structure of eticlopride (**e**)

To explore the TME in Type 1 and Type 2 patients, we conducted cell infiltration analysis. Results revealed significant differences in the proportion of stromal cells and immune cells between Type1 and Type2 patients, including T cells (t = − 4.3, *p* = 1.8e-5), CD8 T cells (t = − 3.6, *p* = 4.0e-4), cytotoxic lymphocytes (t = − 3.9, *p* = 1.2e-4), B cells (t = − 6.7, *p* = 7.1e-11), NK cells (t = − 4.3, *p* = 2.4e-5), monocytes (t = − 5.7, *p* = 2.1e-8), dendritic cells (t = − 8.4, *p* = 6.9e-16), neutrophils (t = − 3.6, p = 4.0e-4), endothelial cells (t = − 13.3, *p* = 9.3e-33) and fibroblasts (t = − 13.7, *p* = 4.1e-34) (Fig. 2a). Subsequently we investigated the relationship between cell proportion and OS, and found that the higher proportion of neutrophils (Fig. 2b) and endothelial cells (Fig. 2c), the poorer the survival of patients. As can be seen from the violin plot, there were significantly fewer neutrophils and endothelial cells in Type1 than in Type2, which suggested that the neutrophils and endothelial cells may play a promoting role in the development and progression of GC, which was also responsible for the poorer survival of Type2 patients. In order to provide a treatment regime for Type2 patients as a reference, differential expression analysis was conducted between Type1 and Type2 patients. Connectivity Map (CMap) analysis was performed using the differentially expressed genes (DEGs) screened out so that we can identify two small molecule drugs that could be used as potential targeted therapeutic drugs for GC (Supplemental Table S4). The chemical structures of these two small molecule drugs were shown. They were thiamine (mean connective score = − 0.735; *P* = 0.00018; Fig. 2d) and eticlopride (mean connective score = − 0.254; *P* = 0.00074; Fig. 2e).

### Construction of prognostically relevant gene set

**Fig. 3:**
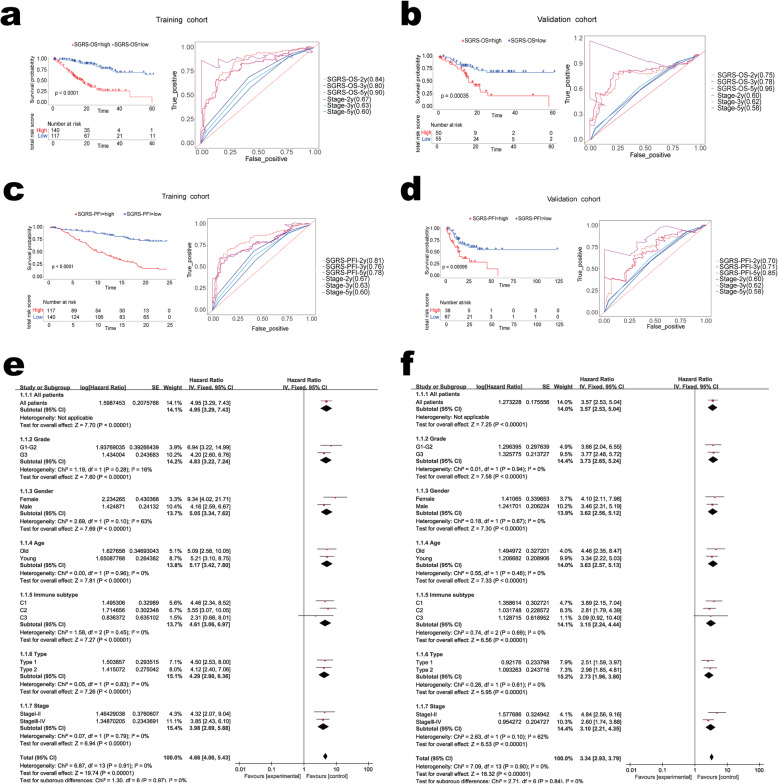
SGRS panel is a prognostic marker. **a**–**b** Kaplan–Meier curves (left) and ROC curves (right) of overall survival according to SGRS-OS groups in the training cohort **a** and validation cohort **b**; **c**-**d** Kaplan–Meier curves (left) and ROC curves (right) of progression-free interval according to SGRS-PFI groups; **e**-**f** Forest plots of the associations between SGRS-OS and overall survival **e**; and the associations between SGRS-PFI and progression-free interval **f** in various subgroups

To establish a reliable model for prognostic prediction, LASSO Cox regression model was utilized to reduce the dimensionality of those 425 stable genes. All cases were classified as the training or the validation cohort according to the stage with a ratio of 7:3 to analyze the prognosis. Differences were not statistically significant in clinical features between groups above (Supplemental Table S1). Through the LASSO model, based on the information OS and PFI, we generated stable gene sets (Supplemental Fig. S1a-d). The OS stable gene set contained 18 genes, and the PFI gene set contained 21 genes (Supplemental Table S5). Then, Cox analysis was performed on the two gene sets to establish two prognostic models respectively. The coefficient of each gene was obtained and stable gene risk scores of OS (SGRS-OS) and PFI were acquired (SGRS-PFI) (Supplemental Table S5). All cases were classified as 2 groups based on SGRS-OS and SGRS-PFI, and the cutoff value calculated by the whole queue was adopted (0.14 for SGRS-OS and 1.44 for SGRS-PFI). In the training and validation sets, the Kaplan Meier curves showed that patients in the high SGRS-OS cohort had a worse prognosis. (Fig. 3a-b). In the ROC, SGRS-OS, which served as the continuous variable in both training and validation cohorts, displayed high predicting ability compared with the TNM classification system. Stage was a categorical variable, so SGRS-OS was converted into a four-categorical variable, for the sake of enhancing the comparability. Even as a categorical variable, the prediction accuracy of SGRS-OS remains good (Supplemental Fig. S2a-b). Similar results were also found for the SGRS-PFI set with documented PFI information (Fig. 3c-d, Supplemental Fig. S2c-d). The predictive ability of SGRS-OS and SGRS-PFI models was tested in each subgroup stratified by immune subtype, grade, sex, stage and age in the whole cohort, respectively, and SGRS-OS and SGRS-PFI were analyzed as continuous variables. As observed from the forest plots, the greater values of the two models markedly identified cases with dismal prognostic outcomes in each subgroup (Fig. 3e-f).

### Stable gene set predicted the efficacy of chemotherapy in GC

**Fig. 4:**
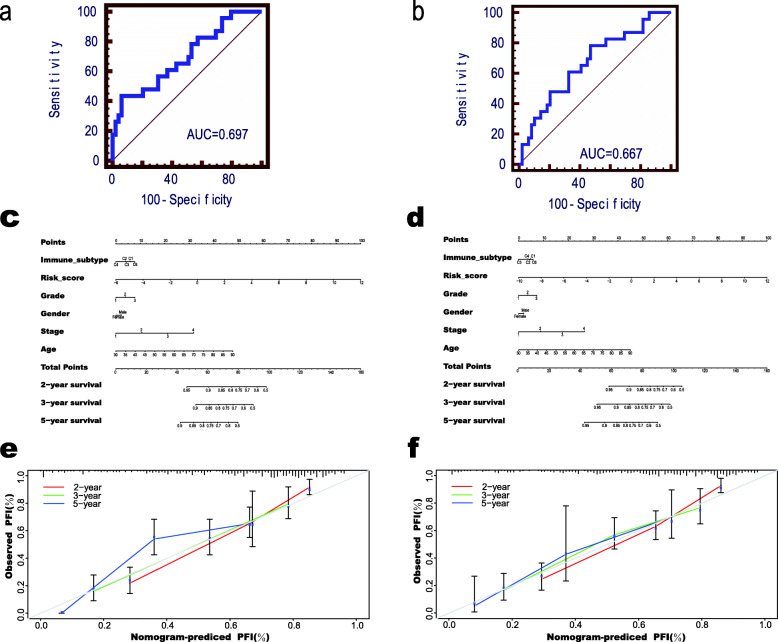
Prediction of the chemotherapy efficacy in gastric cancer. **a** ROC curves of using SGRS-OS to predict the efficacy of chemotherapy; **b** ROC curves of using SGRS-PFI to predict the efficacy of chemotherapy; **c**-**d** Nomograms for predicting the probability of patient mortality based on SGRS-OS **c**, SGRS-PFI **d**, and clinical variables; **e**-**f** The calibration of nomograms based on SGRS-OS **e** and SGRS-PFI **f** in terms of the agreement between predicted and observed 2-year, 3-year, and 5-year outcomes

Relative to supportive care [15], systemic chemotherapy, which is associated with the advantages of survival as well as quality of life, is developed to be the standard therapeutic modality to manage the metastatic or unresectable GC [16]. In order to give clues to conventional chemotherapy regimens, we screened the patients with chemotherapy information and combined the chemotherapy results with SGRS-OS and SGRS-PFI to explore the relationship. The ROC curve showed that low SGRS-OS patients were associated with good chemotherapy outcomes, while high SGRS-OS patients tended to be associated with bad chemotherapy outcomes (Fig. 4a). The same results can be seen when using SGRS-PFI to predict the efficacy of chemotherapy (Fig. 4b). In order to exclude the influence of patients’ own conditions on the prediction of efficacy, we classified the patients according to grade, stage and type, and SGRS-OS and SGRS-PFI were used to predict the chemotherapy efficacy of the patients in every group. In each group, both SGRS-OS and SGRS-PFI were effective predictors of chemotherapy outcomes (Supplemental Fig. S3a-c). We could use the SGRS-OS and SGRS-PFI to predict the chemotherapy efficacy of patients, providing a strong reference for clinical patients to judge the outcomes of chemotherapy. For developing a related quantitative approach to predict the mortality possibility in patients, 2 nomograms were established in the present work, whose C-index were 0.777 and 0.769 respectively (Fig. 4c-d) by enrolling the prognostic factors and scores obtained from the stable gene set. As suggested by the calibration plots, those as-constructed nomograms had favorable performance (Fig. 4e-f).

### Identification of SGRS-OS and SGRS-PFI related clinical characters and biological pathways

**Fig. 5:**
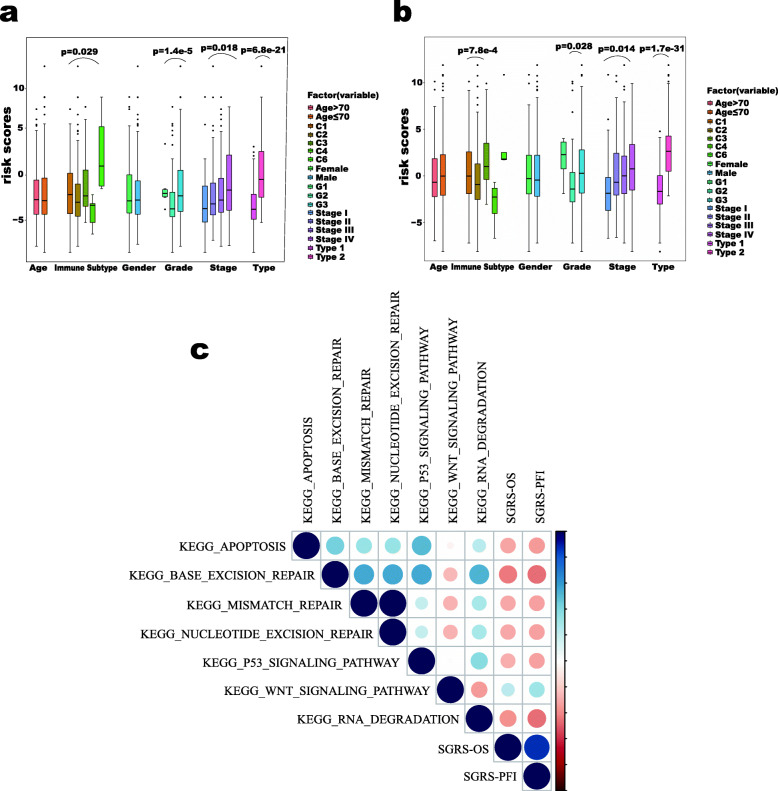
Clinical significance and biological function of SGRS panel. **a**–**b** SGRS-OS values **a** and SGRS-PFI values **b** in different clinical subgroups. Boxes represent the 25–75% of values, blacklines in boxes represent the median values, whiskers represent the 1.5 interquartile ranges, and black dots represent the outliers; **c**Correlation matrix of SGRS-OS, SGRS-PFI values, and biological process activation level. The shaded color indicates the value of the corresponding correlation coefficient, and the area size indicates the *p*-value

This study also examined the correlations between scores obtained from the stable gene set and clinical features/molecular subtypes (Fig. 5a–b). In terms of clinical features, SGRS-OS and SGRS- PFI were significantly increased in more advanced stage patients. Grade also affected the scores of the stable gene set, while age and gender had less influence on the it. In terms of molecular typing, we observed that the SGRS-OS and SGRS-PFI for Type2 patients were also higher than Type1 patients. In terms of the pathway, we found that both SGRS-OS and SGRS-PFI values were significantly correlated with apoptosis, base excision repair and RNA degradation (Fig. 5c). Therefore, higher risk scores tended to be associated with poorer clinical outcomes and tumor-promoting pathways, which provided a strong basis to predict the prognosis of GC.

### Identification of CGB8 as a potential biological target

**Fig. 6:**
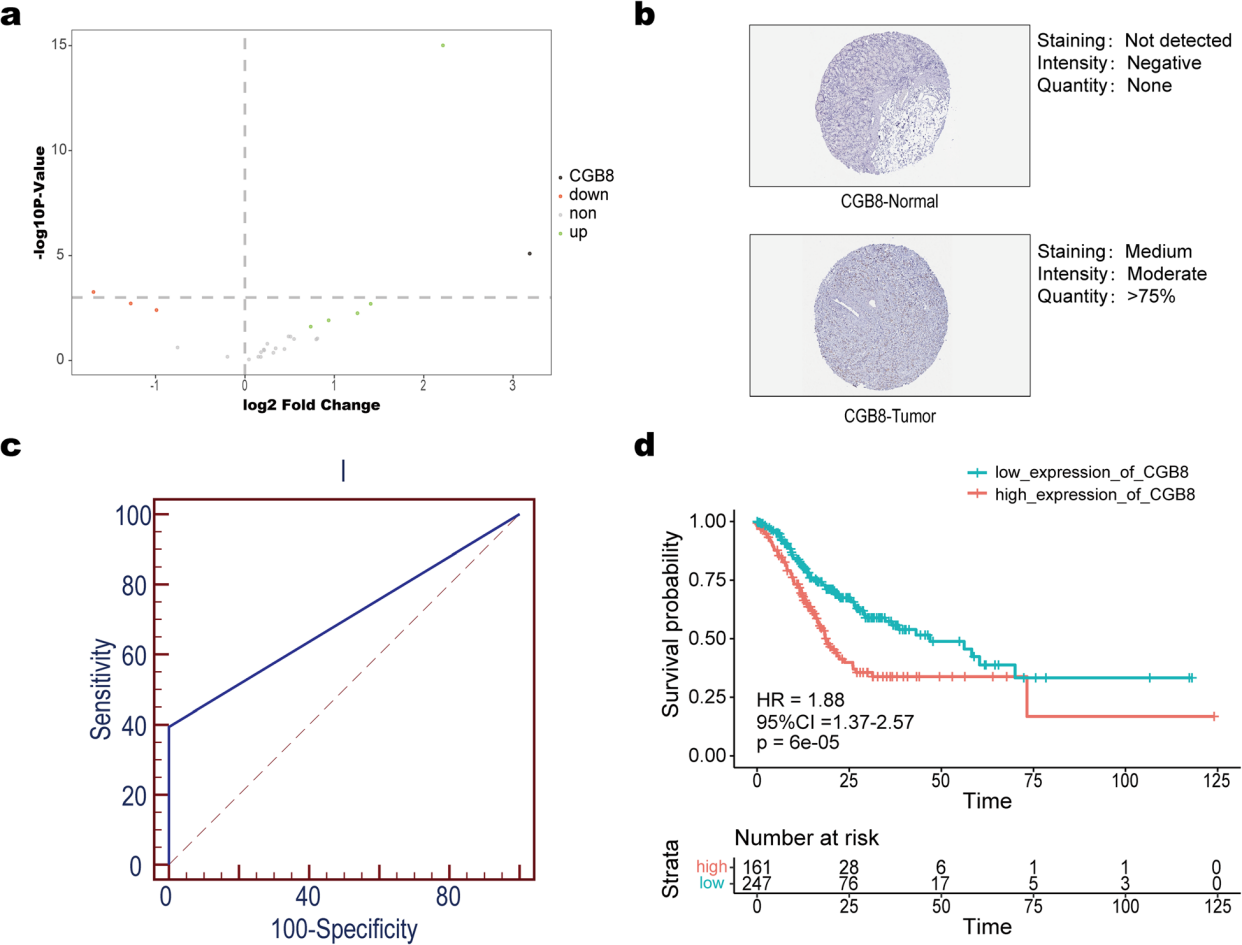
Identification of CGB3 as a potential biological target. **a** Volcano plots of gene expression profiles in TCGA. Using red/green to mark the down/up-regulated genes according to the criteria: *P*-value < 0.05; **b** Immunohistochemistry from the HPA was used to explore expressional deference of CGB8 between normal tissues and gastric cancer tissues; **c** ROC curve analysis of CGB8 in TCGA; **d** Kaplan–Meier curves of overall survival according to the expression levels of CGB8

To provide a target for early diagnosis with GC, differential expression analysis of modeling genes was performed using GC samples and normal samples. 9 DEGs were identified, of which 3 were down-regulated and 6 were up-regulated (Fig. 6a). For better validating the as-constructed stable signature, those 9 DEGs were compared in normal versus GC tissues derived from the HPA. It was suggested by immunohistochemical results that, CGB8 (ENSG00000213030.5) expression upregulated within GC tissues, confirming the difference in CGB8 level in normal versus GC tissues (Fig. 6b). Furthermore, ROC curve analysis was also performed for evaluating CGB8 sensitivity and specificity in diagnosing GC. ROC curves of CGB8 in TCGA database was displayed (Fig. 6c), showing good sensitivity and specificity with AUC of 0.700. In addition, survival analysis showed that CGB8 was a risk factor in the progression of GC (Fig. 6d). Of note, the expression and function of CGB8 in GC remained largely unknown. Therefore, we proposed CGB8 as a biological target and tried to discover its role in GC development.

## Discussion

GC ranks the 6th place in terms of its morbidity within cancer globally, and it is also a major reason for cancer deaths. Although important advances have been made in the molecular mechanism, diagnosis, treatment selection and strategies of tumorigenesis, OS in GC patients still needs to be further improved [17]. The great GC morbidity may be ascribed to the fact that, specific prognostic markers are lacking, which leads to the failure to timely adjust the clinical treatment plan of GC patients [18]. Carbohydrate antigen (CA) 19–9, CA72–4, and carcinoembryonic antigen have been the extensively adopted GC biomarkers, yet they are not the best diagnostic and prognostic biomarkers for GC because of the limited specificity or sensitivity [19, 20]. As a result, it is necessary to identify the novel prognostic biomarkers for GC.

The DNA microarray technique is the efficient biomedical approach at present, and it can be applied in various diagnostic fields [21]. There have been many reports on predicting the prognosis of GC with single gene, but the accuracy of prediction results still needs to be improved [22]. In addition, the prognostic value of Tumor-associated macrophages (TAM) density in GC patients has been analyzed. The results showed that compared with low-density TAM patients, the HR of OS and PFI of high-density TAM patients were 1.56 and 1.10 respectively, indicating that TAM density did not significantly predict adverse survival of GC patients, and TAM density was not an independent predictor of survival of GC patients [23]. Our analysis of cell infiltration showed that there were also significant differences in the composition of stromal cells such as fibroblasts and endothelial cells in Type1 and Type2 patients in addition to immune cells. It can be seen that the number of stromal cells was also an important factor in predicting prognosis of GC, and analyzing the number of immune cells only it is one-sided and inaccurate. Sequencing all human genes is not practical in clinical prediction, but single gene prediction is not accurate enough, so we need to develop an effective gene group for prediction. We performed stability analysis and survival analysis on all genes of GC to screen out the stable prognostic genes. The results of the immune infiltration estimation showed that the genes were related to a variety of immune cells and stromal cells, which were in close connection with the TME, providing a comprehensive view of GC.

We combined the results of molecular typing with the result of infiltration analysis and found that neutrophils and endothelial cells were strongly associated with prognosis. An increase in neutrophils and endothelial cells often predicts a worse prognosis. Neutrophil levels have been shown to be a strong predictor of poor survival in GC patients. In patients with GC, accumulation of peripheral blood and invasive marginal neutrophils promotes disease progression and predicts poor survival [24]. In addition, studies have shown that endothelial cells such as lymphatic endothelial cells and vascular endothelial cells can promote the metastasis or growth of GC [25, 26]. The accuracy of our results is further verified. Treatment regimens targeting neutrophils and endothelial cells may improve the patient’s condition.

We used LASSO Cox regression to screen the optimal combination of genes and establish two models, called SGRS-OS and SGRS- PFI. The two models contained 18 and 21 genes, respectively. We can predict the prognosis of patients with GC, timely adjust our treatment plan, to maximize the survival of patients. In the future, the development of a kit to test this gene set could promote the clinical prognosis prediction of GC for the benefit of mankind.

CGB8 was proposed as a biological target in our study. This gene is a member of the glycoprotein hormone beta chain family and encodes the beta 8 subunit of chorionic gonadotropin (CG). Recent studies have shown that CGB8 could also be used as an immune-related prognostic model gene for oral squamous cell carcinoma (OSCC) [27]. Combined with our analysis, in conclusion, CGB8 had the ability to diagnose GC and predict tumor prognosis to a certain extent. It is hoped that this study can provide support for future exploration of CGB8. Further studying of selected genes, we found that some genes such as PLA2R1, GPC3, AKR1B1 and SERPINE1B were closely related to the TME. Some reports find that PLA2R1 is expressed in neutrophils [28] and pulmonary macrophages [29]. Additionally, PLA2R1 is able to enhance the tumor suppressing responses, such as apoptosis, senescence, or transformation suppression. PLA2R1 is down-regulated in a number of cancer types, which supports its tumor suppressor role, and its expression can be suppressed by c-MYC and HIF2α, the oncogenes [30]. Additionally, GPC3, one of the tumor-associated antigens, elevated F4/80 + CD86+ macrophage (M1) percentage within tumor, in the meantime of inducing CD8+ T cell immune response specific to GPC3 [31]. Fidarestat, an inhibitor of AKR1B1, can markedly suppress the inflammatory signals induced by growth factors, tumor necrosis factor-alpha (TNF-α), environmental allergens, and lipopolysachharide (LPS), and such signals may result in various inflammatory disorders. The inflammatory disorder animal model like cardiovascular disease (CVD), diabetes, metastasis, uveitis, cancer and asthma, inhibiting AKR1B1 evidently promotes disease occurrence [32]. SERPINE1B is associated with B cell function [33]. These genes related to the TME were selected and involved in modeling that greatly improving the accuracy of the model. Although these have been proved to be closely related to tumor, there were still few studies related to GC. Our findings provide new ideas and methods for searching for potential biological targets of GC.

Generally, 80–90% GC cases are diagnosed at the advanced stage when the cancer cannot be resected or may relapse or metastasize after surgery [17, 34]. Although molecular targeted therapy is promising for improving the survival of patients with advanced GC, due to the high heterogeneity of GC and the lack of targets, fewer patients receive appropriate molecular targeted therapy. Therefore, systemic chemotherapy is still the main treatment method for patients with advanced GC [35]. Therefore, prediction of chemotherapy outcomes is crucial to the formulation of patient prognosis and improvement of patient survival. We found significant differences in the efficacy of chemotherapy in different patients with SGRS-OS. The chemotherapy efficacy of patients with low SGRS-OS was significantly better than that of patients with low SGRS-OS, suggesting a correlation between the two. The results of SGRS-OS can be used to predict the chemotherapy efficacy of patients with good accuracy. This method is expected to solve the problem that prognosis of GC is difficult to predict.

This study had some limitations. Firstly, the patient population was heterogeneous. Secondly, we used the patients in the TCGA dataset to model. Some modeling genes were not found in the patient expression matrix in the GEO dataset. Therefore, we did not use a validation set from the GEO database. Special attention should be paid when using the stable gene set to detect patients in other databases. Thirdly, the gene expression data were imported to the Cox regression model as categorical variables in this work. Therefore, more studies are needed to verify the optimal thresholds.

To sum up, our constructed stable gene set can stably predict patients’ prognosis, guide the treatment for GC patients and has a good prospect of clinical application.

## Supplementary Information

**Additional file 1: Supplemental Table S1**. Patients’ basic characteristics.

**Additional file 2: Supplemental Table S2**. Stable prognostic genes.

**Additional file 3: Supplemental Table S3**. Clinical characteristics of two types*.

**Additional file 4: Supplemental Table S5**. CMap analysis results.

**Additional file 5: Supplemental Table S4**. Stable genes of model.

**Additional file 6: Supplemental Fig. S1**. Feature selection for building SGRS. (a-b) Ten-time cross-validation for tuning parameter selection in the LASSO model. Solid vertical lines represent partial likelihood deviance ± standard error (SE). The dotted vertical lines are drawn at the optimal values by minimum criteria and 1-SE criteria. The partial likelihood deviance versus log (λ) was plotted, where λ is the tuning parameter. Using LASSO to select genes based on the information OS (left) and PFI (right). (c-d) LASSO coefficient profiles of the 425 selected features are presented based on the information OS (left) and PFI (right).

**Additional file 7: Supplemental Fig. S2**. Predictive accuracy of the SGRS panel as category variables. (a-b) SGRS-OS was fourfold classified based on cut-off values. Kaplan–Meier curves (left) and ROC curves (right) of OS according to SGRS-OS groups in the training cohort. (c-d) SGRS-PFI was fourfold classified based on cut-off values calculated. Kaplan–Meier curves (left) and ROC curves (right) of PFI according to SGRS-PFI groups in the training cohort.

**Additional file 8: Supplemental Fig. S3**. SGRS-OS and SGRS-PFI were used to predict the chemotherapy efficacy of the patients in every group. (a-c) SGRS-OS was used to predict the efficacy of chemotherapy and drawing the ROC curves (a-c) in different groups divided by grades (a), stages (b), and types (c); (d-f) SGRS-PFI was used to predict the efficacy of chemotherapy and drawing the ROC curves (d-f) in different groups divided by grade (d), stage (e), and type (f).

## Data Availability

The datasets used and/or analyzed during the current study are available from the TCGA. The URL link and the accession number of the data used from the TCGA database is: https://xenabrowser.net/
