# Supplementary material for: A stable gene set for prediction of prognosis and efficacy of chemotherapy in gastric cancer
Source: BMC Cancer. 2021 Jun 10;21:684. doi: 10.1186/s12885-021-08444-w (PMC8194165; doi:10.1186/s12885-021-08444-w)
Supplement: Supplementary file 1 — Additional file 1: Supplemental Table S1. Patients’ basic characteristics. [file 12885_2021_8444_MOESM1_ESM.docx]

**Supplemental Table S1. Patients’ basic characteristics**

| **Item** | **Entire cohort** | **Random grouping** | | |
| --- | --- | --- | --- | --- |
|  |  | **Training cohort** | **Validation cohort** | **p-value** |
| **Age**  (mean ± SD) | 65.15 ± 10.71 | 64.88 ± 11.01 | 65.83 ± 9.91 | 0.43 |
| **Gender**(n, %) |  |  |  | 0.69 |
| Male | 234 (64.6) | 164 (63.8) | 70 (66.7) |  |
| Female | 128 (35.4) | 93 (36.2) | 35 (33.3) |  |
| **Stage** (n, %) |  |  |  | 0.57 |
| Stage _ low | 166 (45.8) | 118 (46.0) | 48 (45.7) |  |
| Stage _ high | 196 (54.2) | 139 (54.0) | 66 (54.3) |  |
| **Grade**(n, %) |  |  |  | >0.9 |
| G _ low | 136 (37.6) | 97 (37.7) | 39 (37.1) |  |
| G _ high | 226 (62.4) | 160 (62.3) | 66 (62.9) |  |
| Abbreviation: *SD, standard deviation* | | | | |
