# Supplementary material for: A stable gene set for prediction of prognosis and efficacy of chemotherapy in gastric cancer
Source: BMC Cancer. 2021 Jun 10;21:684. doi: 10.1186/s12885-021-08444-w (PMC8194165; doi:10.1186/s12885-021-08444-w)
Supplement: Supplementary file 2 — Additional file 2: Supplemental Table S2. Stable prognostic genes. [file 12885_2021_8444_MOESM2_ESM.docx]

**Supplemental Table S2 stable prognostic genes**

| **ID** | **Gene** | **Times for significance** |
| --- | --- | --- |
| 1 | ENSG00000106366.8 | 1000 |
| 2 | ENSG00000228623.3 | 1000 |
| 3 | ENSG00000254738.1 | 1000 |
| 4 | ENSG00000255622.3 | 1000 |
| 5 | ENSG00000258511.1 | 1000 |
| 6 | ENSG00000280021.1 | 1000 |
| 7 | ENSG00000205562.2 | 999 |
| 8 | ENSG00000227757.3 | 999 |
| 9 | ENSG00000234821.1 | 999 |
| 10 | ENSG00000236847.1 | 999 |
| 11 | ENSG00000249472.1 | 999 |
| 12 | ENSG00000165694.9 | 998 |
| 13 | ENSG00000229847.8 | 998 |
| 14 | ENSG00000278309.1 | 998 |
| 15 | ENSG00000197023.6 | 997 |
| 16 | ENSG00000244968.6 | 997 |
| 17 | ENSG00000255268.2 | 997 |
| 18 | ENSG00000279847.3 | 997 |
| 19 | ENSG00000152402.10 | 996 |
| 20 | ENSG00000188069.4 | 996 |
| 21 | ENSG00000232759.1 | 996 |
| 22 | ENSG00000256343.7 | 996 |
| 23 | ENSG00000054803.3 | 995 |
| 24 | ENSG00000134548.9 | 995 |
| 25 | ENSG00000166450.12 | 995 |
| 26 | ENSG00000132031.12 | 994 |
| 27 | ENSG00000184258.6 | 994 |
| 28 | ENSG00000251283.1 | 994 |
| 29 | ENSG00000179676.6 | 993 |
| 30 | ENSG00000227511.1 | 993 |
| 31 | ENSG00000261122.6 | 993 |
| 32 | ENSG00000131095.11 | 992 |
| 33 | ENSG00000213030.5 | 992 |
| 34 | ENSG00000223168.1 | 992 |
| 35 | ENSG00000223917.1 | 992 |
| 36 | ENSG00000235620.1 | 992 |
| 37 | ENSG00000275620.1 | 992 |
| 38 | ENSG00000101276.14 | 991 |
| 39 | ENSG00000147257.13 | 991 |
| 40 | ENSG00000170370.11 | 991 |
| 41 | ENSG00000227585.2 | 991 |
| 42 | ENSG00000242009.1 | 991 |
| 43 | ENSG00000257639.1 | 990 |
| 44 | ENSG00000259363.5 | 990 |
| 45 | ENSG00000101327.8 | 989 |
| 46 | ENSG00000229892.1 | 989 |
| 47 | ENSG00000236719.2 | 989 |
| 48 | ENSG00000277631.4 | 989 |
| 49 | ENSG00000004846.16 | 988 |
| 50 | ENSG00000169302.14 | 988 |
| 51 | ENSG00000254728.1 | 988 |
| 52 | ENSG00000270852.1 | 988 |
| 53 | ENSG00000139117.13 | 987 |
| 54 | ENSG00000136487.17 | 986 |
| 55 | ENSG00000099250.17 | 985 |
| 56 | ENSG00000139910.19 | 985 |
| 57 | ENSG00000225519.1 | 985 |
| 58 | ENSG00000236333.3 | 985 |
| 59 | ENSG00000277499.1 | 985 |
| 60 | ENSG00000158560.14 | 983 |
| 61 | ENSG00000175175.5 | 983 |
| 62 | ENSG00000250835.1 | 983 |
| 63 | ENSG00000113209.8 | 982 |
| 64 | ENSG00000229242.1 | 982 |
| 65 | ENSG00000260105.6 | 982 |
| 66 | ENSG00000004948.13 | 981 |
| 67 | ENSG00000105825.11 | 980 |
| 68 | ENSG00000130844.16 | 980 |
| 69 | ENSG00000182836.9 | 980 |
| 70 | ENSG00000140873.15 | 979 |
| 71 | ENSG00000224210.1 | 979 |
| 72 | ENSG00000225147.1 | 979 |
| 73 | ENSG00000233824.1 | 979 |
| 74 | ENSG00000238249.2 | 979 |
| 75 | ENSG00000274525.1 | 979 |
| 76 | ENSG00000113211.5 | 978 |
| 77 | ENSG00000134376.14 | 978 |
| 78 | ENSG00000267172.1 | 978 |
| 79 | ENSG00000272108.1 | 978 |
| 80 | ENSG00000113361.12 | 977 |
| 81 | ENSG00000203971.1 | 977 |
| 82 | ENSG00000227423.2 | 976 |
| 83 | ENSG00000162267.12 | 975 |
| 84 | ENSG00000169169.14 | 975 |
| 85 | ENSG00000229063.2 | 975 |
| 86 | ENSG00000081818.3 | 974 |
| 87 | ENSG00000085662.13 | 974 |
| 88 | ENSG00000243978.8 | 974 |
| 89 | ENSG00000165495.15 | 973 |
| 90 | ENSG00000171495.16 | 973 |
| 91 | ENSG00000182916.7 | 973 |
| 92 | ENSG00000227467.3 | 973 |
| 93 | ENSG00000268603.1 | 973 |
| 94 | ENSG00000273940.1 | 973 |
| 95 | ENSG00000276562.1 | 973 |
| 96 | ENSG00000171502.14 | 972 |
| 97 | ENSG00000205090.8 | 972 |
| 98 | ENSG00000261051.1 | 971 |
| 99 | ENSG00000269964.2 | 971 |
| 100 | ENSG00000143028.8 | 970 |
| 101 | ENSG00000228776.2 | 970 |
| 102 | ENSG00000236373.1 | 970 |
| 103 | ENSG00000266127.1 | 970 |
| 104 | ENSG00000099875.14 | 969 |
| 105 | ENSG00000184227.7 | 969 |
| 106 | ENSG00000232471.3 | 969 |
| 107 | ENSG00000265190.6 | 969 |
| 108 | ENSG00000152284.4 | 968 |
| 109 | ENSG00000039560.13 | 967 |
| 110 | ENSG00000154545.16 | 967 |
| 111 | ENSG00000171444.17 | 967 |
| 112 | ENSG00000279581.1 | 967 |
| 113 | ENSG00000258294.5 | 966 |
| 114 | ENSG00000273923.1 | 966 |
| 115 | ENSG00000164318.17 | 965 |
| 116 | ENSG00000185985.8 | 965 |
| 117 | ENSG00000224127.1 | 965 |
| 118 | ENSG00000227880.1 | 965 |
| 119 | ENSG00000249247.1 | 965 |
| 120 | ENSG00000249452.2 | 965 |
| 121 | ENSG00000260343.1 | 965 |
| 122 | ENSG00000133067.17 | 964 |
| 123 | ENSG00000267583.5 | 964 |
| 124 | ENSG00000171643.13 | 963 |
| 125 | ENSG00000246695.7 | 963 |
| 126 | ENSG00000106436.4 | 962 |
| 127 | ENSG00000219139.1 | 962 |
| 128 | ENSG00000226813.2 | 962 |
| 129 | ENSG00000233640.2 | 962 |
| 130 | ENSG00000259509.2 | 962 |
| 131 | ENSG00000259979.1 | 962 |
| 132 | ENSG00000125879.4 | 961 |
| 133 | ENSG00000197445.2 | 961 |
| 134 | ENSG00000260679.1 | 961 |
| 135 | ENSG00000004799.7 | 960 |
| 136 | ENSG00000065325.12 | 960 |
| 137 | ENSG00000180745.4 | 960 |
| 138 | ENSG00000226314.7 | 960 |
| 139 | ENSG00000249152.1 | 960 |
| 140 | ENSG00000255053.1 | 960 |
| 141 | ENSG00000255349.1 | 960 |
| 142 | ENSG00000144031.11 | 959 |
| 143 | ENSG00000166478.9 | 959 |
| 144 | ENSG00000186119.7 | 959 |
| 145 | ENSG00000254038.1 | 959 |
| 146 | ENSG00000255426.1 | 959 |
| 147 | ENSG00000267141.1 | 959 |
| 148 | ENSG00000276141.4 | 959 |
| 149 | ENSG00000189058.8 | 957 |
| 150 | ENSG00000127990.15 | 956 |
| 151 | ENSG00000226329.2 | 956 |
| 152 | ENSG00000238004.1 | 956 |
| 153 | ENSG00000135299.16 | 955 |
| 154 | ENSG00000185818.7 | 955 |
| 155 | ENSG00000053918.15 | 954 |
| 156 | ENSG00000170891.10 | 954 |
| 157 | ENSG00000235151.1 | 954 |
| 158 | ENSG00000239344.1 | 954 |
| 159 | ENSG00000267145.1 | 954 |
| 160 | ENSG00000108381.10 | 953 |
| 161 | ENSG00000198739.10 | 953 |
| 162 | ENSG00000234882.1 | 953 |
| 163 | ENSG00000248172.1 | 953 |
| 164 | ENSG00000269706.1 | 953 |
| 165 | ENSG00000271486.1 | 953 |
| 166 | ENSG00000219736.1 | 952 |
| 167 | ENSG00000261253.2 | 952 |
| 168 | ENSG00000280073.1 | 952 |
| 169 | ENSG00000121966.6 | 951 |
| 170 | ENSG00000162687.16 | 951 |
| 171 | ENSG00000181984.11 | 951 |
| 172 | ENSG00000221870.2 | 951 |
| 173 | ENSG00000230975.1 | 951 |
| 174 | ENSG00000146938.14 | 950 |
| 175 | ENSG00000218410.1 | 950 |
| 176 | ENSG00000272937.1 | 950 |
| 177 | ENSG00000279249.1 | 949 |
| 178 | ENSG00000265544.1 | 948 |
| 179 | ENSG00000179083.6 | 947 |
| 180 | ENSG00000215117.5 | 947 |
| 181 | ENSG00000260478.1 | 947 |
| 182 | ENSG00000131016.16 | 946 |
| 183 | ENSG00000186684.12 | 946 |
| 184 | ENSG00000196408.11 | 946 |
| 185 | ENSG00000224657.9 | 946 |
| 186 | ENSG00000228935.1 | 946 |
| 187 | ENSG00000258721.2 | 946 |
| 188 | ENSG00000198691.11 | 945 |
| 189 | ENSG00000259283.2 | 945 |
| 190 | ENSG00000260223.1 | 944 |
| 191 | ENSG00000141854.7 | 943 |
| 192 | ENSG00000214022.11 | 943 |
| 193 | ENSG00000215846.6 | 943 |
| 194 | ENSG00000231541.1 | 943 |
| 195 | ENSG00000243877.1 | 943 |
| 196 | ENSG00000253925.1 | 943 |
| 197 | ENSG00000275367.1 | 943 |
| 198 | ENSG00000091656.15 | 942 |
| 199 | ENSG00000171016.11 | 942 |
| 200 | ENSG00000184408.9 | 942 |
| 201 | ENSG00000234393.1 | 942 |
| 202 | ENSG00000249396.1 | 942 |
| 203 | ENSG00000100478.14 | 941 |
| 204 | ENSG00000224807.5 | 941 |
| 205 | ENSG00000224922.1 | 941 |
| 206 | ENSG00000279118.1 | 941 |
| 207 | ENSG00000279751.1 | 941 |
| 208 | ENSG00000241808.1 | 940 |
| 209 | ENSG00000091128.12 | 939 |
| 210 | ENSG00000141338.13 | 939 |
| 211 | ENSG00000223536.5 | 939 |
| 212 | ENSG00000261190.5 | 939 |
| 213 | ENSG00000267097.1 | 939 |
| 214 | ENSG00000138448.11 | 938 |
| 215 | ENSG00000226739.1 | 938 |
| 216 | ENSG00000257580.1 | 938 |
| 217 | ENSG00000186971.3 | 937 |
| 218 | ENSG00000243378.1 | 937 |
| 219 | ENSG00000164128.6 | 936 |
| 220 | ENSG00000234810.1 | 936 |
| 221 | ENSG00000254986.7 | 936 |
| 222 | ENSG00000057019.15 | 935 |
| 223 | ENSG00000169105.7 | 935 |
| 224 | ENSG00000182348.6 | 935 |
| 225 | ENSG00000204918.3 | 935 |
| 226 | ENSG00000204919.1 | 935 |
| 227 | ENSG00000227151.4 | 935 |
| 228 | ENSG00000234278.3 | 935 |
| 229 | ENSG00000241992.3 | 934 |
| 230 | ENSG00000246430.6 | 934 |
| 231 | ENSG00000267528.1 | 934 |
| 232 | ENSG00000279460.1 | 934 |
| 233 | ENSG00000281720.1 | 934 |
| 234 | ENSG00000110203.8 | 933 |
| 235 | ENSG00000179097.5 | 933 |
| 236 | ENSG00000203739.3 | 933 |
| 237 | ENSG00000250573.1 | 933 |
| 238 | ENSG00000259107.1 | 932 |
| 239 | ENSG00000113083.12 | 931 |
| 240 | ENSG00000135318.11 | 931 |
| 241 | ENSG00000138674.16 | 931 |
| 242 | ENSG00000166073.8 | 931 |
| 243 | ENSG00000236854.1 | 931 |
| 244 | ENSG00000261763.1 | 931 |
| 245 | ENSG00000279526.1 | 931 |
| 246 | ENSG00000230469.1 | 930 |
| 247 | ENSG00000236897.1 | 930 |
| 248 | ENSG00000244259.1 | 930 |
| 249 | ENSG00000257476.1 | 930 |
| 250 | ENSG00000264300.1 | 930 |
| 251 | ENSG00000163114.5 | 929 |
| 252 | ENSG00000179046.8 | 929 |
| 253 | ENSG00000229824.1 | 929 |
| 254 | ENSG00000230687.1 | 929 |
| 255 | ENSG00000234257.1 | 929 |
| 256 | ENSG00000270385.1 | 929 |
| 257 | ENSG00000232968.1 | 928 |
| 258 | ENSG00000166363.5 | 927 |
| 259 | ENSG00000205189.11 | 927 |
| 260 | ENSG00000233477.1 | 927 |
| 261 | ENSG00000250992.1 | 927 |
| 262 | ENSG00000258683.1 | 927 |
| 263 | ENSG00000134533.6 | 926 |
| 264 | ENSG00000141447.16 | 926 |
| 265 | ENSG00000224465.1 | 926 |
| 266 | ENSG00000233211.2 | 926 |
| 267 | ENSG00000254993.1 | 926 |
| 268 | ENSG00000277654.4 | 926 |
| 269 | ENSG00000279455.1 | 926 |
| 270 | ENSG00000166068.12 | 925 |
| 271 | ENSG00000182983.14 | 925 |
| 272 | ENSG00000226671.1 | 925 |
| 273 | ENSG00000240545.3 | 925 |
| 274 | ENSG00000249501.1 | 925 |
| 275 | ENSG00000256684.1 | 925 |
| 276 | ENSG00000270997.1 | 925 |
| 277 | ENSG00000109113.17 | 924 |
| 278 | ENSG00000187243.16 | 924 |
| 279 | ENSG00000233620.5 | 924 |
| 280 | ENSG00000266875.2 | 924 |
| 281 | ENSG00000233105.1 | 923 |
| 282 | ENSG00000038427.15 | 922 |
| 283 | ENSG00000167670.15 | 922 |
| 284 | ENSG00000184394.1 | 922 |
| 285 | ENSG00000237565.3 | 922 |
| 286 | ENSG00000254895.2 | 922 |
| 287 | ENSG00000257519.1 | 922 |
| 288 | ENSG00000259620.1 | 920 |
| 289 | ENSG00000265240.1 | 920 |
| 290 | ENSG00000214518.3 | 919 |
| 291 | ENSG00000264659.1 | 919 |
| 292 | ENSG00000065320.8 | 917 |
| 293 | ENSG00000188833.9 | 917 |
| 294 | ENSG00000225110.2 | 917 |
| 295 | ENSG00000241722.2 | 917 |
| 296 | ENSG00000100121.12 | 916 |
| 297 | ENSG00000153246.11 | 916 |
| 298 | ENSG00000168350.7 | 916 |
| 299 | ENSG00000227062.2 | 916 |
| 300 | ENSG00000259245.1 | 916 |
| 301 | ENSG00000116741.7 | 915 |
| 302 | ENSG00000226567.1 | 915 |
| 303 | ENSG00000085117.11 | 914 |
| 304 | ENSG00000167641.10 | 914 |
| 305 | ENSG00000214020.3 | 914 |
| 306 | ENSG00000232749.2 | 914 |
| 307 | ENSG00000126838.9 | 913 |
| 308 | ENSG00000255580.1 | 913 |
| 309 | ENSG00000260338.1 | 913 |
| 310 | ENSG00000204583.9 | 912 |
| 311 | ENSG00000258496.1 | 912 |
| 312 | ENSG00000130224.14 | 911 |
| 313 | ENSG00000144366.15 | 911 |
| 314 | ENSG00000236965.4 | 911 |
| 315 | ENSG00000248869.5 | 911 |
| 316 | ENSG00000272569.1 | 911 |
| 317 | ENSG00000131386.17 | 910 |
| 318 | ENSG00000225971.1 | 910 |
| 319 | ENSG00000236858.1 | 910 |
| 320 | ENSG00000253484.1 | 910 |
| 321 | ENSG00000254891.1 | 910 |
| 322 | ENSG00000184911.14 | 909 |
| 323 | ENSG00000203685.9 | 909 |
| 324 | ENSG00000225253.1 | 909 |
| 325 | ENSG00000230531.1 | 909 |
| 326 | ENSG00000248365.3 | 909 |
| 327 | ENSG00000197134.11 | 907 |
| 328 | ENSG00000242615.1 | 907 |
| 329 | ENSG00000249274.1 | 907 |
| 330 | ENSG00000249484.8 | 907 |
| 331 | ENSG00000229788.1 | 906 |
| 332 | ENSG00000261496.1 | 905 |
| 333 | ENSG00000251345.2 | 904 |
| 334 | ENSG00000255244.1 | 904 |
| 335 | ENSG00000277558.1 | 904 |
| 336 | ENSG00000279542.1 | 904 |
| 337 | ENSG00000225238.1 | 903 |
| 338 | ENSG00000261711.6 | 903 |
| 339 | ENSG00000265099.1 | 903 |
| 340 | ENSG00000117152.13 | 902 |
| 341 | ENSG00000134986.13 | 902 |
| 342 | ENSG00000157152.16 | 902 |
| 343 | ENSG00000224435.2 | 902 |
| 344 | ENSG00000251452.2 | 902 |
| 345 | ENSG00000230879.1 | 901 |
| 346 | ENSG00000268549.1 | 901 |
| 347 | ENSG00000260042.1 | 900 |
| 348 | ENSG00000265101.1 | 899 |
| 349 | ENSG00000282591.1 | 899 |
| 350 | ENSG00000216713.1 | 898 |
| 351 | ENSG00000233787.3 | 898 |
| 352 | ENSG00000223626.1 | 897 |
| 353 | ENSG00000234481.1 | 897 |
| 354 | ENSG00000121904.17 | 896 |
| 355 | ENSG00000186393.5 | 896 |
| 356 | ENSG00000235136.1 | 895 |
| 357 | ENSG00000224549.1 | 893 |
| 358 | ENSG00000258965.2 | 892 |
| 359 | ENSG00000171408.13 | 891 |
| 360 | ENSG00000184302.6 | 891 |
| 361 | ENSG00000279367.1 | 891 |
| 362 | ENSG00000123360.11 | 890 |
| 363 | ENSG00000213548.3 | 890 |
| 364 | ENSG00000251483.2 | 890 |
| 365 | ENSG00000198948.11 | 889 |
| 366 | ENSG00000232500.1 | 889 |
| 367 | ENSG00000223978.1 | 888 |
| 368 | ENSG00000226616.1 | 888 |
| 369 | ENSG00000215034.4 | 887 |
| 370 | ENSG00000223368.2 | 887 |
| 371 | ENSG00000244392.3 | 887 |
| 372 | ENSG00000167825.3 | 886 |
| 373 | ENSG00000227547.3 | 886 |
| 374 | ENSG00000280143.1 | 886 |
| 375 | ENSG00000113249.12 | 885 |
| 376 | ENSG00000261101.2 | 885 |
| 377 | ENSG00000123119.11 | 884 |
| 378 | ENSG00000224189.6 | 883 |
| 379 | ENSG00000250993.1 | 883 |
| 380 | ENSG00000223995.2 | 882 |
| 381 | ENSG00000250003.2 | 882 |
| 382 | ENSG00000118903.6 | 881 |
| 383 | ENSG00000174099.10 | 880 |
| 384 | ENSG00000184954.4 | 880 |
| 385 | ENSG00000252484.1 | 879 |
| 386 | ENSG00000276632.1 | 878 |
| 387 | ENSG00000216721.3 | 877 |
| 388 | ENSG00000230788.2 | 870 |
| 389 | ENSG00000234878.1 | 870 |
| 390 | ENSG00000248029.2 | 870 |
| 391 | ENSG00000260268.5 | 870 |
| 392 | ENSG00000126106.13 | 869 |
| 393 | ENSG00000243165.1 | 869 |
| 394 | ENSG00000225444.1 | 868 |
| 395 | ENSG00000176904.1 | 865 |
| 396 | ENSG00000224043.7 | 865 |
| 397 | ENSG00000253304.1 | 865 |
| 398 | ENSG00000158270.11 | 864 |
| 399 | ENSG00000184351.7 | 864 |
| 400 | ENSG00000234560.3 | 863 |
| 401 | ENSG00000272168.5 | 863 |
| 402 | ENSG00000253371.1 | 862 |
| 403 | ENSG00000213908.4 | 856 |
| 404 | ENSG00000248346.1 | 856 |
| 405 | ENSG00000278727.1 | 856 |
| 406 | ENSG00000254407.1 | 855 |
| 407 | ENSG00000215206.5 | 853 |
| 408 | ENSG00000248684.1 | 852 |
| 409 | ENSG00000228853.1 | 848 |
| 410 | ENSG00000233036.1 | 846 |
| 411 | ENSG00000215353.2 | 845 |
| 412 | ENSG00000232799.1 | 845 |
| 413 | ENSG00000259417.2 | 843 |
| 414 | ENSG00000219757.1 | 840 |
| 415 | ENSG00000249698.1 | 840 |
| 416 | ENSG00000131477.10 | 835 |
| 417 | ENSG00000248185.1 | 835 |
| 418 | ENSG00000145861.7 | 832 |
| 419 | ENSG00000254228.1 | 832 |
| 420 | ENSG00000254487.2 | 831 |
| 421 | ENSG00000251003.7 | 822 |
| 422 | ENSG00000249945.1 | 815 |
| 423 | ENSG00000270661.1 | 807 |
| 424 | ENSG00000242120.3 | 784 |
| 425 | ENSG00000213312.3 | 770 |
