# Supplementary material for: A stable gene set for prediction of prognosis and efficacy of chemotherapy in gastric cancer
Source: BMC Cancer. 2021 Jun 10;21:684. doi: 10.1186/s12885-021-08444-w (PMC8194165; doi:10.1186/s12885-021-08444-w)
Supplement: Supplementary file 3 — Additional file 3: Supplemental Table S3. Clinical characteristics of two types*. [file 12885_2021_8444_MOESM3_ESM.docx]

**Supplemental Table S3. Clinical characteristics of two types***

| **Item** |  | **molecular subgroups using robust prognostic genes (%)** | | |
| --- | --- | --- | --- | --- |
|  |  | **Type1** | **Type2** | **p-value** |
| **Stage**  (n, %) | Stage_low | 99 (47.4) | 53 (42.4) | 0.44 |
|  | Stage_high | 110 (52.6) | 72 (57.6) |  |
| **Age**  (n, %) | Old | 73 (34.9) | 39 (31.2) | 0.56 |
|  | Young | 136 (65.1) | 86 (68.8) |  |
| **Gender**  (n, %) | Male | 139 (66.5) | 82 (65.6) | >0.9 |
|  | Female | 70(33.5) | 43 (34.4) |  |
| **Grade**  (n, %) | G_low | 98 (46.9) | 29 (23.2) | 2.7e-5 |
|  | G_high | 111 (53.1) | 96 (76.8) |  |
| **Immune subtype** (n, %) | C1 | 68 (32.5) | 44 (35.2) | 2.59e-8 |
|  | C2 | 129 (61.7) | 50 (40.0) |  |
|  | C3 | 5(2.4) | 26 (20.8) |  |
|  | C4 | 6 (2.9) | 1 (0.8) |  |
|  | C6 | 1 (0.5) | 4 (3.2) |  |
| **CMS** (n, %) | GI.CIN | 115 (55.3) | 77 (61.6) | 1.4e-9 |
|  | GI.EBV | 23 (11.0) | 7 (5.6) |  |
|  | GI.GS | 12 (5.8) | 33 (26.4) |  |
|  | GI.HM-indel | 54 (26.0) | 6 (4.8) |  |
|  | GI.HM-SNV | 4 (1.9) | 2 (1.6) |  |
| **The number of samples containing the existing immune subtype and TCGA clustering information was 334.* | | | | |
