# Supplementary material for: A stable gene set for prediction of prognosis and efficacy of chemotherapy in gastric cancer
Source: BMC Cancer. 2021 Jun 10;21:684. doi: 10.1186/s12885-021-08444-w (PMC8194165; doi:10.1186/s12885-021-08444-w)
Supplement: Supplementary file 4 — Additional file 4: Supplemental Table S5. CMap analysis results. [file 12885_2021_8444_MOESM4_ESM.docx]

**Supplemental Table S5 CMap analysis results**

| **Cmap name** | **Mean connective score** | **n** | **Enrichment** | **P-value** | **Specificity** | **Percent non-null** |
| --- | --- | --- | --- | --- | --- | --- |
| puromycin | 0.805 | 4 | 0.943 | 0 | 0.0449 | 100 |
| thiamine | -0.735 | 3 | -0.961 | 0.00018 | 0 | 100 |
