# Supplementary material for: A stable gene set for prediction of prognosis and efficacy of chemotherapy in gastric cancer
Source: BMC Cancer. 2021 Jun 10;21:684. doi: 10.1186/s12885-021-08444-w (PMC8194165; doi:10.1186/s12885-021-08444-w)
Supplement: Supplementary file 5 — Additional file 5: Supplemental Table S4. Stable genes of model. [file 12885_2021_8444_MOESM5_ESM.docx]

**Supplemental Table S4. Stable genes of model**

| **Stable genes of OS model** | | | |
| --- | --- | --- | --- |
| **Rank** | **Gene** | **Symbol** | **Coefficients** |
| 1 | ENSG00000085662.13 | AKR1B1 | 0.2325768 |
| 2 | ENSG00000100121.12 | GGTLC2 | 0.18403808 |
| 3 | ENSG00000106366.8 | SERPINE1 | 0.04335621 |
| 4 | ENSG00000106436.4 | MYL10 | 0.18174387 |
| 5 | ENSG00000125879.4 | OTOR | 0.05447163 |
| 6 | ENSG00000153246.11 | PLA2R1 | 0.26850997 |
| 7 | ENSG00000171495.16 | MROH2B | 0.18080842 |
| 8 | ENSG00000179676.6 | LINC00305 | 0.17474755 |
| 9 | ENSG00000213030.5 | CGB8 | 0.1168756 |
| 10 | ENSG00000214518.3 | KRTAP2-2 | 0.11239258 |
| 11 | ENSG00000234821.1 | MRPS33P4 | 0.15525434 |
| 12 | ENSG00000236719.2 | OVAAL | 0.12718008 |
| 13 | ENSG00000239344.1 | AC090686.1 | 0.0138728 |
| 14 | ENSG00000251452.2 | AC109811.2 | 0.33360805 |
| 15 | ENSG00000257639.1 | AC005632.1 | 0.15949701 |
| 16 | ENSG00000258511.1 | LINC02295 | 0.21523218 |
| 17 | ENSG00000260679.1 | AP007216.2 | 0.14680921 |
| 18 | ENSG00000279847.3 | C8orf87 | 0.05368559 |

| **Stable genes of PFI model** | | | |
| --- | --- | --- | --- |
| **Rank** | **Gene** | **Symbol** | **Coefficients** |
| 1 | ENSG00000085662.13 | AKR1B1 | 0.103293458 |
| 2 | ENSG00000100121.12 | GGTLC2 | 0.115138763 |
| 3 | ENSG00000106366.8 | SERPINE1 | -0.020959714 |
| 4 | ENSG00000106436.4 | MYL10 | 0.122266283 |
| 5 | ENSG00000126838.9 | PZP | 0.155361958 |
| 6 | ENSG00000147257.13 | GPC3 | 0.146083269 |
| 7 | ENSG00000153246.11 | PLA2R1 | 0.179117713 |
| 8 | ENSG00000157152.16 | SYN2 | 0.073273383 |
| 9 | ENSG00000179676.6 | LINC00305 | 0.08748825 |
| 10 | ENSG00000213030.5 | CGB8 | 0.386672912 |
| 11 | ENSG00000214518.3 | KRTAP2-2 | 0.173750055 |
| 12 | ENSG00000226329.2 | AC005682.1 | 0.026536818 |
| 13 | ENSG00000232759.1 | AC002480.1 | 0.095907567 |
| 14 | ENSG00000234821.1 | MRPS33P4 | 0.092202762 |
| 15 | ENSG00000236719.2 | OVAAL | 0.152781227 |
| 16 | ENSG00000243165.1 | AC025033.1 | 0.079466783 |
| 17 | ENSG00000251452.2 | AC109811.2 | 0.181730581 |
| 18 | ENSG00000258511.1 | LINC02295 | 0.101091402 |
| 19 | ENSG00000260679.1 | AP007216.2 | 0.153027348 |
| 20 | ENSG00000265099.1 | AC090774.1 | -0.008978698 |
| 21 | ENSG00000279542.1 | AC015908.5 | 0.026824837 |
