# Supplementary figures and images for: A stable gene set for prediction of prognosis and efficacy of chemotherapy in gastric cancer
Source: BMC Cancer. 2021 Jun 10;21:684. doi: 10.1186/s12885-021-08444-w (PMC8194165; doi:10.1186/s12885-021-08444-w)

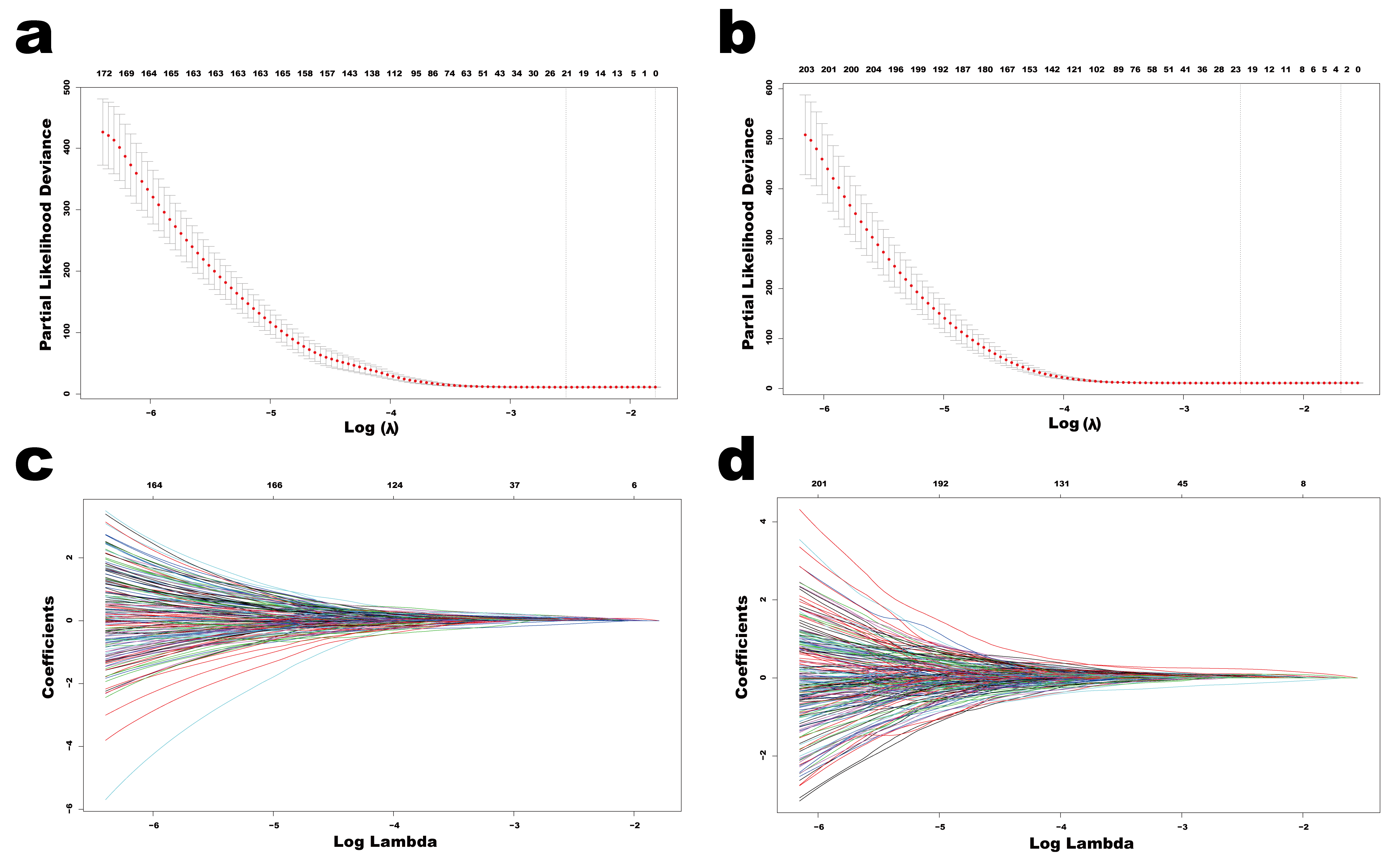

Supplement: Supplementary file 6 — Additional file 6: Supplemental Fig. S1. Feature selection for building SGRS. (a-b) Ten-time cross-validation for tuning parameter selection in the LASSO model. Solid vertical lines represent partial likelihood deviance ± standard error (SE). The dotted vertical lines are drawn at the optimal values by minimum criteria and 1-SE criteria. The partial likelihood deviance versus log (λ) was plotted, where λ is the tuning parameter. Using LASSO to select genes based on the information OS (left) and PFI (right). (c-d) LASSO coefficient profiles of the 425 selected features are presented based on the information OS (left) and PFI (right). [file 12885_2021_8444_MOESM6_ESM.tif]

**a**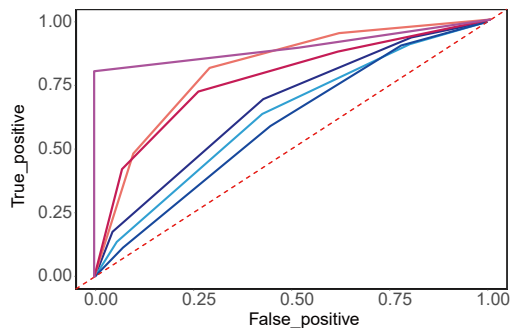**b**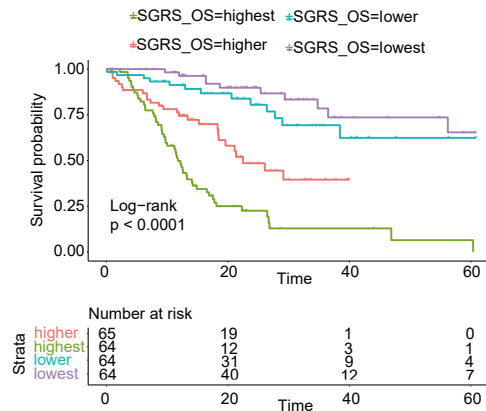**c**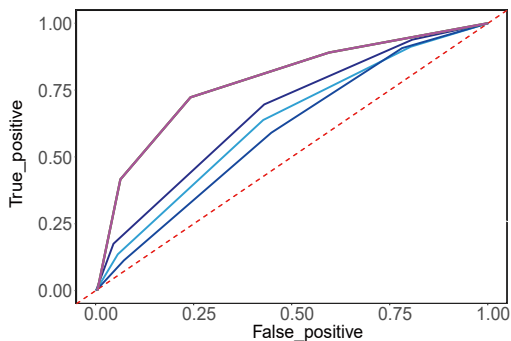**d**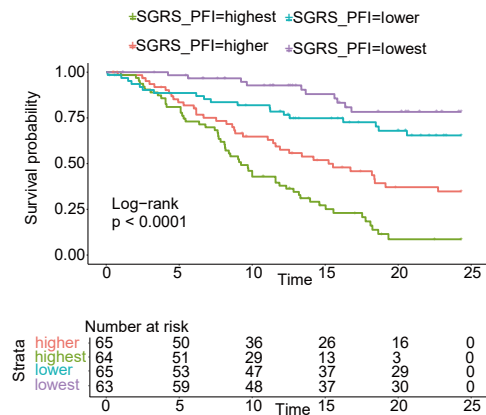

Supplement: Supplementary file 7 — Additional file 7: Supplemental Fig. S2. Predictive accuracy of the SGRS panel as category variables. (a-b) SGRS-OS was fourfold classified based on cut-off values. Kaplan–Meier curves (left) and ROC curves (right) of OS according to SGRS-OS groups in the training cohort. (c-d) SGRS-PFI was fourfold classified based on cut-off values calculated. Kaplan–Meier curves (left) and ROC curves (right) of PFI according to SGRS-PFI groups in the training cohort. [file 12885_2021_8444_MOESM7_ESM.pdf]

## SGRS-OS

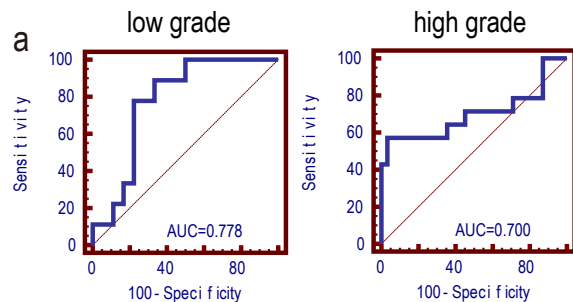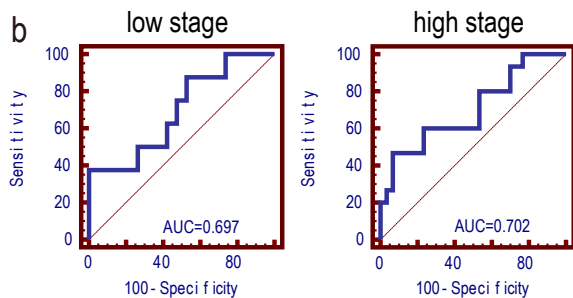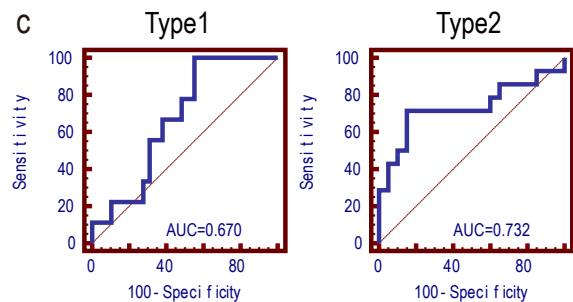

## SGRS-PFI

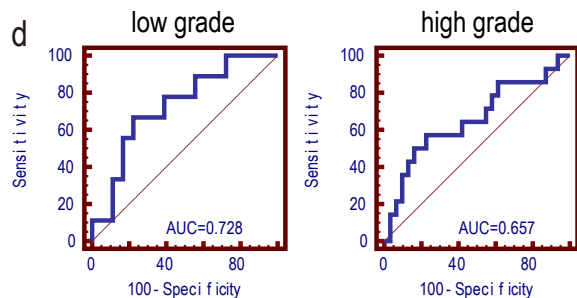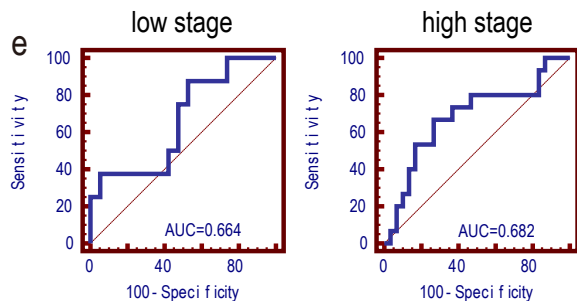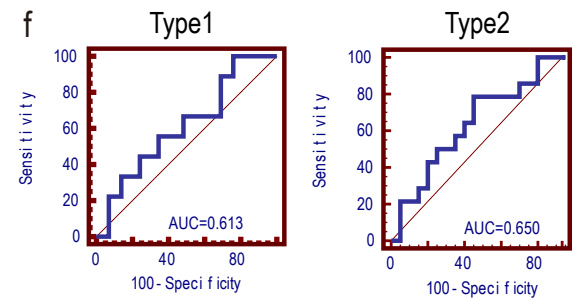

Supplement: Supplementary file 8 — Additional file 8: Supplemental Fig. S3. SGRS-OS and SGRS-PFI were used to predict the chemotherapy efficacy of the patients in every group. (a-c) SGRS-OS was used to predict the efficacy of chemotherapy and drawing the ROC curves (a-c) in different groups divided by grades (a), stages (b), and types (c); (d-f) SGRS-PFI was used to predict the efficacy of chemotherapy and drawing the ROC curves (d-f) in different groups divided by grade (d), stage (e), and type (f). [file 12885_2021_8444_MOESM8_ESM.pdf]
